# Supplementary material for: Experience-dependent structural plasticity targets dynamic filopodia in regulating dendrite maturation and synaptogenesis
Source: Nat Commun. 2018 Aug 22;9:3362. doi: 10.1038/s41467-018-05871-5 (PMC6105721; doi:10.1038/s41467-018-05871-5)
Supplement: Supplementary file 1 — Supplementary Information [file 41467_2018_5871_MOESM1_ESM.pdf]

Supplementary Information

Experience-dependent structural plasticity targets dynamic filopodia in regulating dendrite maturation and synaptogenesis

Sheng et al.

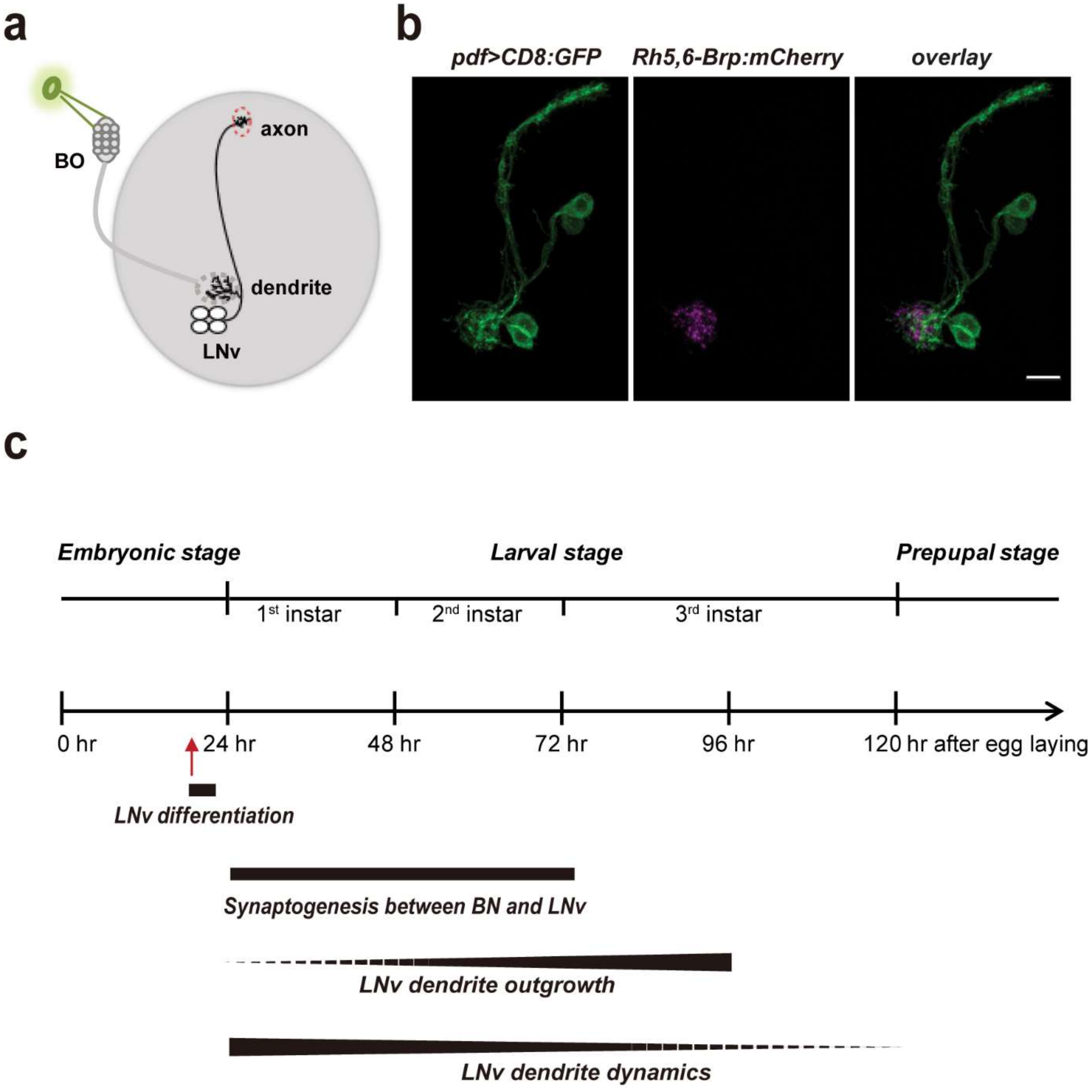

Supplementary Figure 1 The stages of LNv dendrite development. **a** Schematic diagram illustrating the synaptic connections between the Bolwig's organ (BO) and LNvs. The light-sensing BO sends axonal projections into the larval brain and forms synaptic connections with LNv dendrites (dashed gray circle). The LNvs' axon projects to the dorsal part of larval brain (dashed red circle). **b** Representative projected confocal images show the whole profile of LNvs in the larval brain lobe at the late 3rd instar stage. The morphology of LNvs was visualized by pdf-Gal4 driven mCD8:GFP. Presynaptic contacts from BO photoreceptors were labeled by *Rh5,6-Brp:mCherry*. Scale bar, 20 µm. **c** A timeline of the progression of LNv dendrite growth and maturation during larval development. LNv differentiation occurs late in the embryonic stage, at around 18-22hr after egg laying (AEL). Synaptic contacts between the LNv and Bolwig's nerve (BN) are saturated at 72 hr AEL, corresponding to the decline of LNv dendrite dynamics. The expansion of the LNv dendrite continues and plateaus at 96 hr.

a

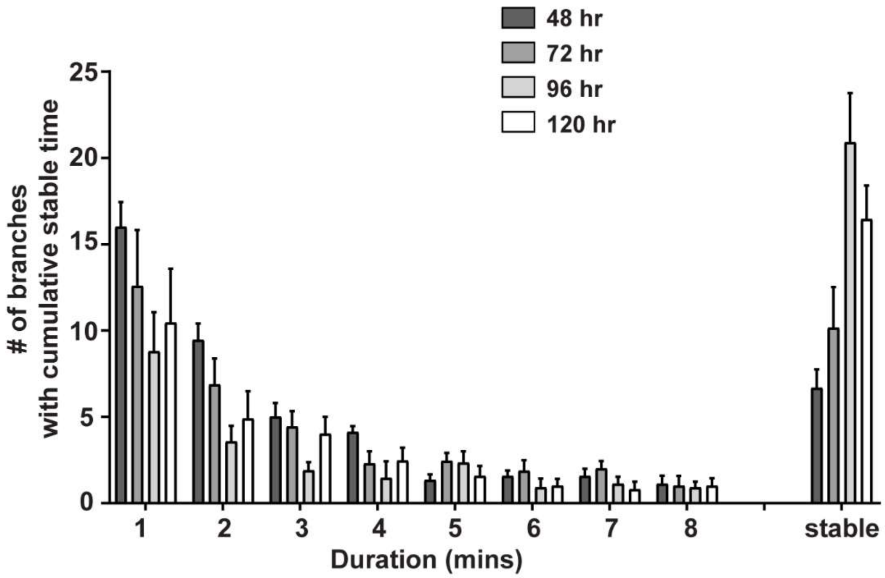

b

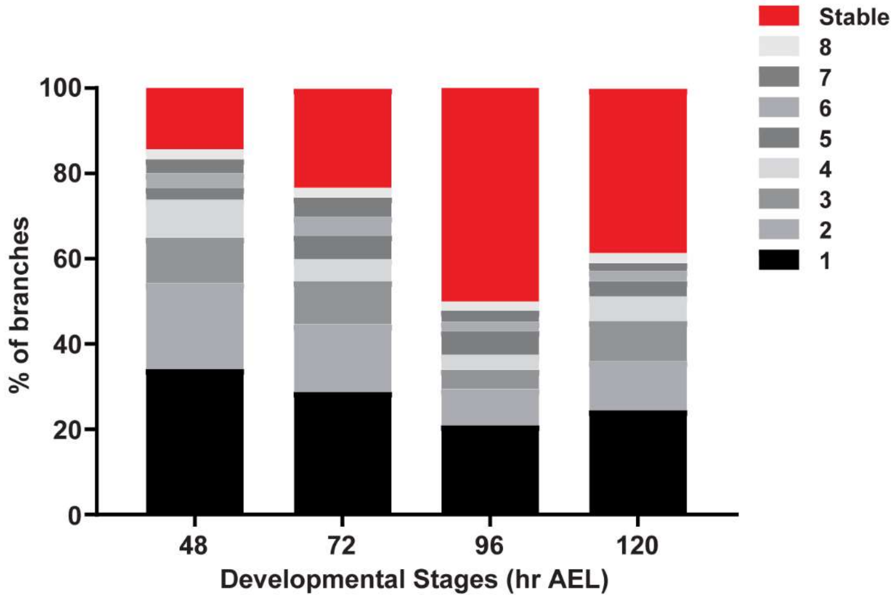

Supplementary Figure 2 The composition of LNv dendritic arbors changes during development**. a** The numbers of dynamic branches that exhibit 1 to 8 min cumulative duration of stableness are plotted together with the numbers of stable branches. Bar heights are means and error bars represent the SEM. **b** The distribution of branches that exhibit different durations of transient stableness (gray, 1-8 min; red, >8 min, stable) at different developmental stages. Stable branches are branches that exhibit no dynamic behavior within 8 min. n = 9, 7, 9 and 9 single-labeled neurons for 48, 72, 96 and 120 hr AEL respectively.

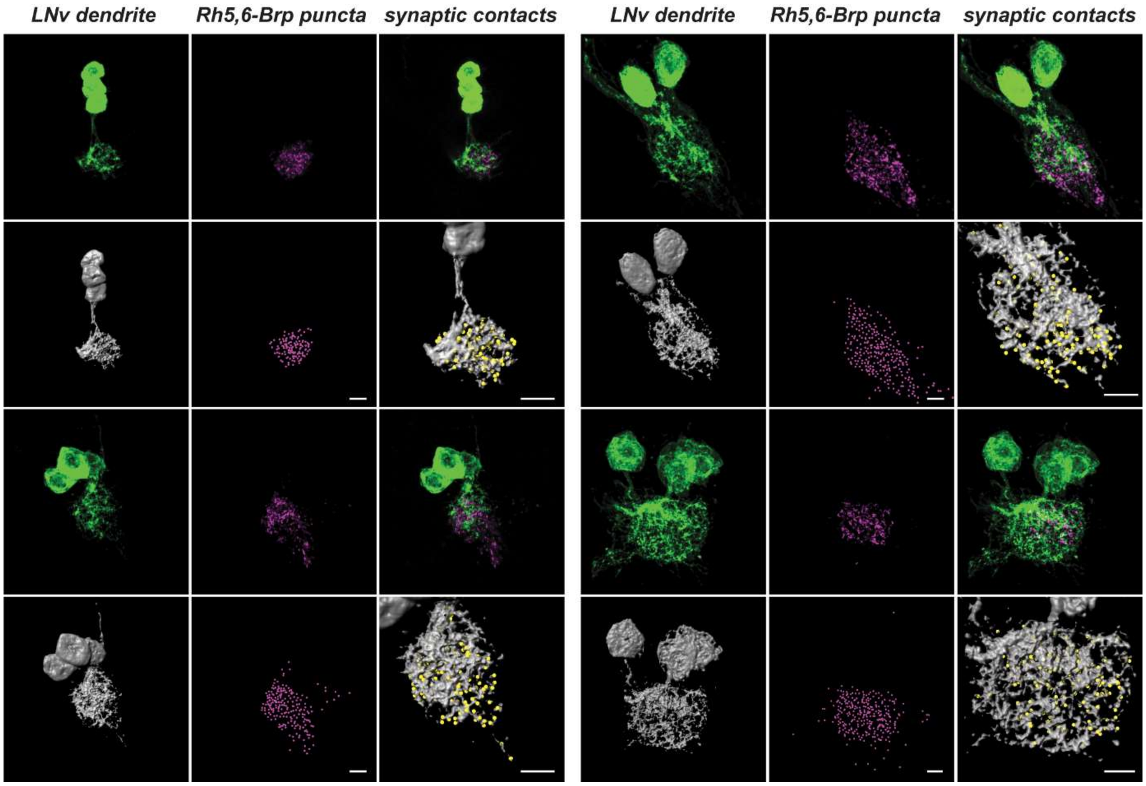

Supplementary Figure 3 Temporal coupling of dendrite dynamics and synaptogenesis in LNvs. Top panels: Representative projected confocal images of the LNv dendrite (green) co-labeled with *Rh5,6-Brp:mCherry* (magenta) collected at 48, 72, 96 and 120 hr AEL are shown. Bottom panels: LNv dendrite volume (gray), the total number of BN synaptic contacts in the larval optic neuropil (LON) (*Rh5,6-Brp:mCherry* puncta, magenta spots) and the number of Brp puncta contacting the LNv dendrites (synaptic contacts, yellow spots) were analyzed using 3D reconstructions. Scale bar, 5 μm.

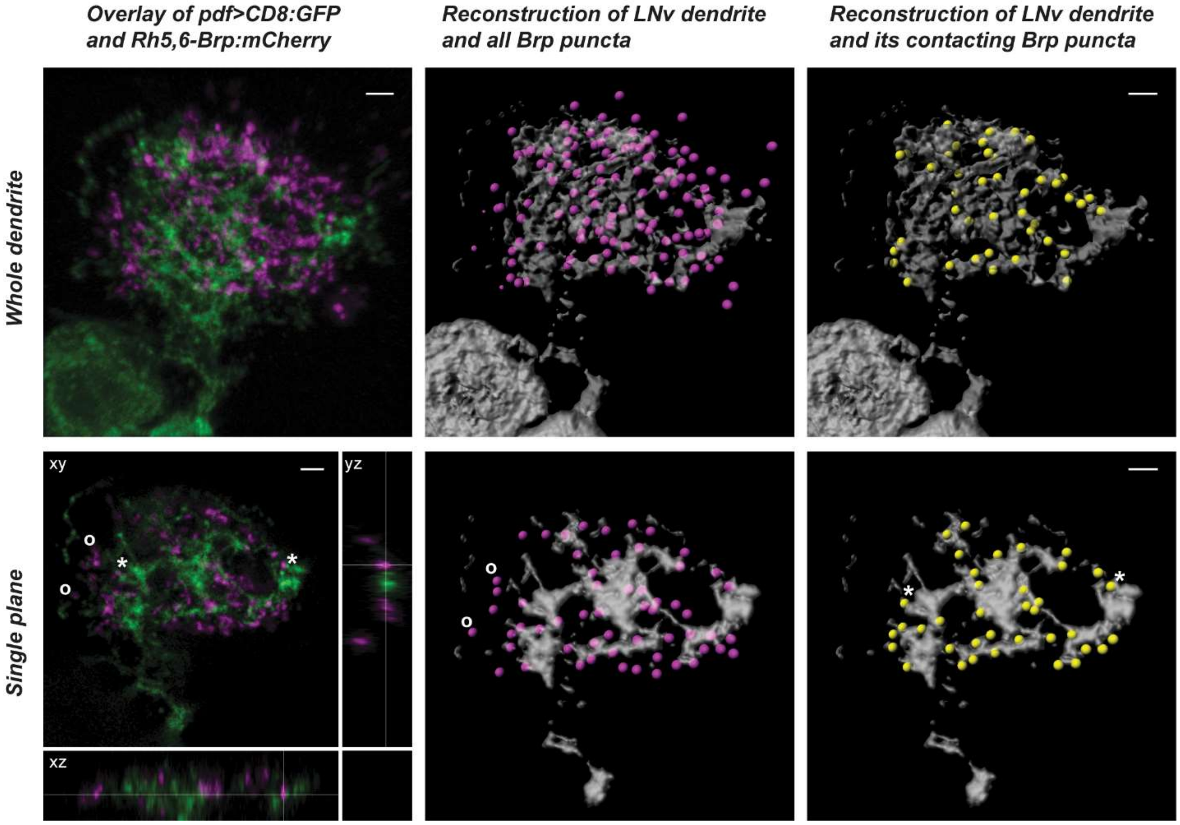

Supplementary Figure 4 3D reconstruction of LNv dendrite volume and putative synaptic contacts. Top panels, a representative projected confocal image (left) and the 3D volume and spot reconstructions (middle) for LNv dendrites (gray) and BN presynaptic contacts (magenta) in the LON are shown. The putative synaptic contacts, the *Rh5,6-Brp* puncta, contacting the LNv dendrite are represented by yellow spots (right). Bottom panels, a single optic section (left) and its reconstruction (middle and right) illustrate the representative Brp puncta contacting LNv dendrite, indicated by *. Two representative non-contacting Brp puncta are indicated by o. Scale bar, 2 µm.

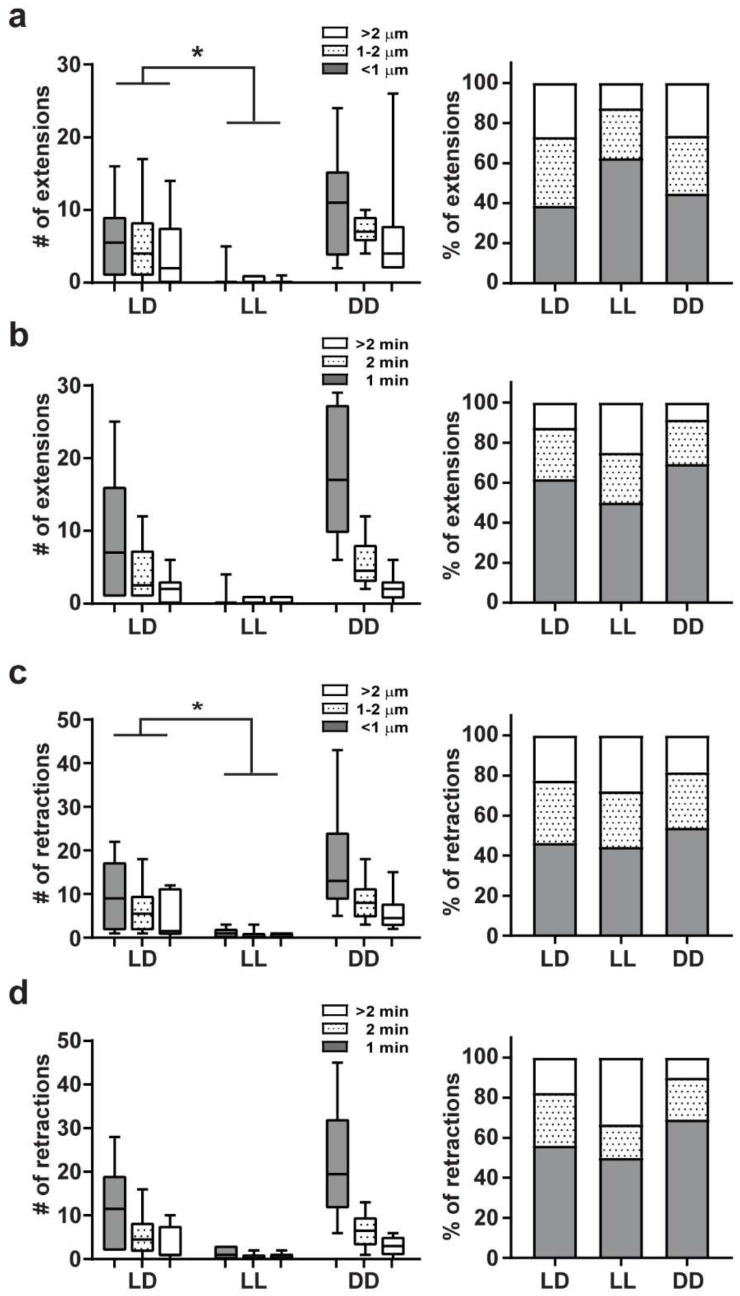

Supplementary Figure 5 Experience-dependent modifications of the dynamic state of LNv dendrites at 120 hr AEL. Light: dark conditions strongly influence LNv dendrite dynamics, reflected by the number of extension **a-b** and retraction **c-d** events. Both were short and transient in all conditions. The distribution of the distance traveled **a**, **c** or the distribution of the duration of the events **b**, **d** with the culture conditions are shown. Statistical significance was assessed by one-way ANOVA with Dunnett post hoc test. Number of extension events **a** ANOVA: $F_{2, 24} = 8.493$, $P = 0.002$; Dunnett: $P = 0.04$, LD vs LL. Number of retraction events **c** ANOVA: $F_{2, 24} = 7.377$, $P = 0.003$; Dunnett: $P = 0.044$, LD vs LL. n = 10, 7 and 10 single-labeled LNvs for LD, LL and DD, respectively. Data are presented as a box plot showing the median (center line in box), interquartile ranges (top and bottom of box, 25–75%) and total ranges (whiskers, 0–100%) for left panels and as a histogram plot for right panels.

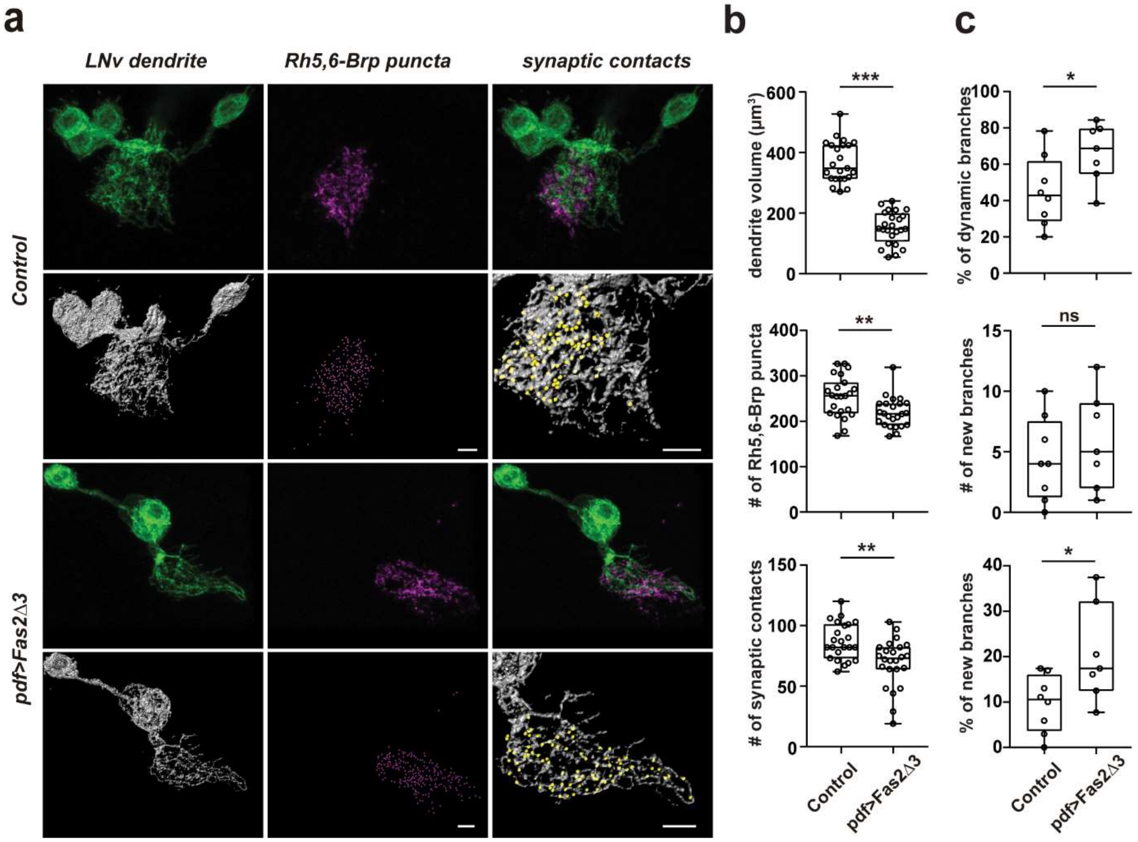

Supplementary Figure 6 Reducing synaptic Fas2 leads to changes in dendrite volume, synaptic contact and dendrite dynamics. Expressing a dominant negative form of the Fas2 transgene (Fas2Δ3) in LNvs reduces the dendrite volume and the number of synaptic contacts while eleavating dendrite dynamics. **a-b** Compared to the background control, expression of Fas2Δ3 in LNvs causes significant reductions of dendrite volume (green or gray), the number of presynaptic terminals (magenta) and synaptic contacts (yellow spots). Representative projected confocal images (Top) or 3D reconstructions (Bottom) are shown. Genotypes are as indicated. Scale bar, 5 µm. **c** Expressing Fas2Δ3 in LNvs increases dendrite dynamics as reflected by the percentage of dynamic branches (Top). There were no signicant change in the total number of newly appeared branches (Middle). However, after normalizing to the number of total branches, Fas2Δ3 expression generates more newly appeared branches (Bottom). Data are presented as a box plot overlaid with a dot plot; the box plot shows the median (center line in box), interquartile ranges (top and bottom of box, 25–75%) and total ranges (whiskers, 0–100%) and the dot plot displays individual data points.

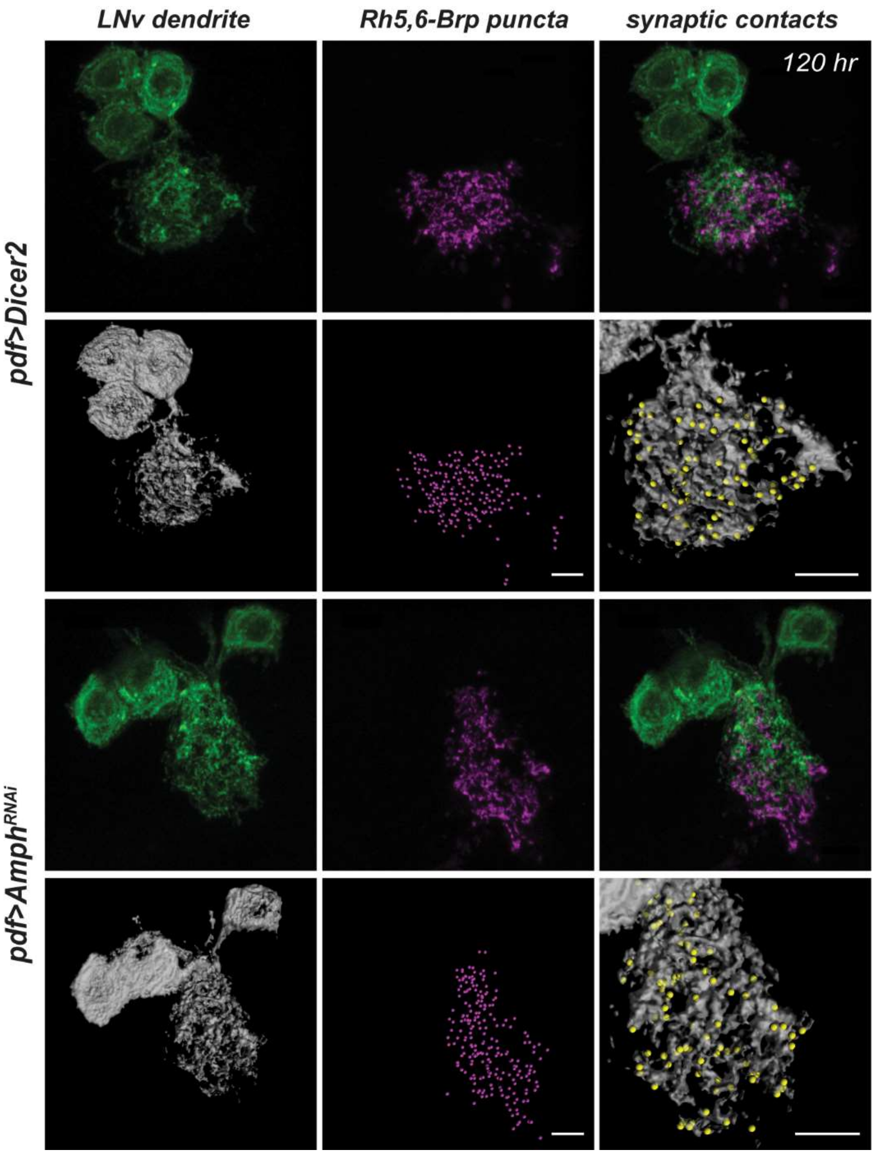

Supplementary Figure 7 Knocking-down Amph in LNvs does not affect the dendrite volume or the number of synaptic contacts. Compared to the control, knocking down Amph in LNvs shows no significant change of dendrite volume (green or gray), the number of presynaptic terminals (magenta) or synaptic contacts (yellow spots). Representative projected confocal images (Top) or 3D reconstructions (Bottom) are shown. Genotypes are as indicated. Scale bar, 5 μm.

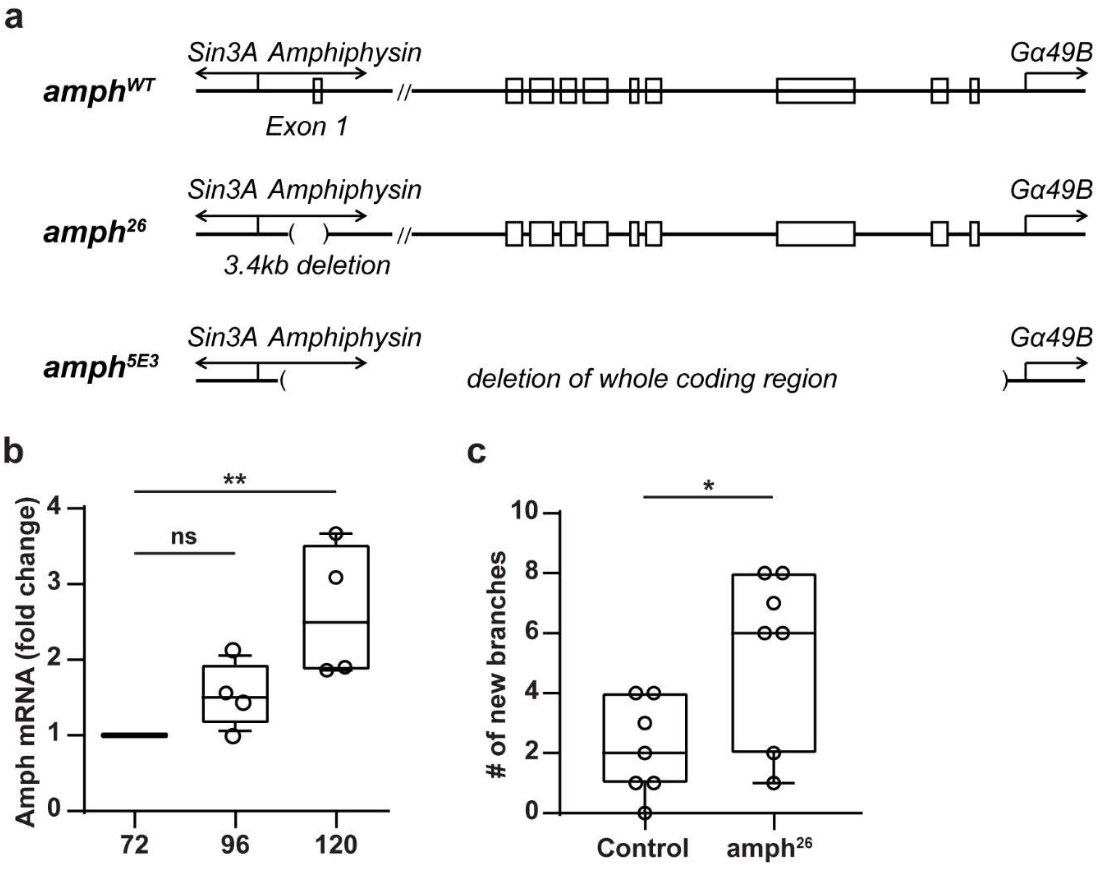

Supplementary Figure 8 Genetic mutants of *amph* show changes in LNv dendrite dynamics. **a** The *amph* gene diagram shows two loss-of-function mutants with deletions eliminating either the start codon or the whole coding sequences of *amph*. **b** Increased Amph expression at 120 hr as compared to 72 hr AEL was revealed by quantitative real-time PCR with three technical replicates for all samples. Results were confirmed in four repeated experiments. Statistical significance was assessed by one-way ANOVA with Dunnett post hoc test. ANOVA: $F_{2, 9} = 8.55$, $P = 0.008$; Dunnett: $P = 0.352$, 72 vs 96 hr; $P = 0.005$, 72 vs 120 hr. **c** LNvs collected from amph^{26} mutants show an increased number of newly formed branches at 120 hr AEL in LL conditions as compared to the controls. n = 7 for both groups. Quantification was from all 4 LNvs of larvae raised in LL to 120 hr AEL. Statistical significance was assessed by unpaired *t* test, $t_{12} = 2.692$, $P = 0.02$. Data are presented as a box plot overlaid with a dot plot; the box plot shows the median (center line in box), interquartile ranges (top and bottom of box, 25–75%) and total ranges (whiskers, 0–100%) and the dot plot displays individual data points. ns, not significant; $*P < 0.05$, $**P < 0.01$.

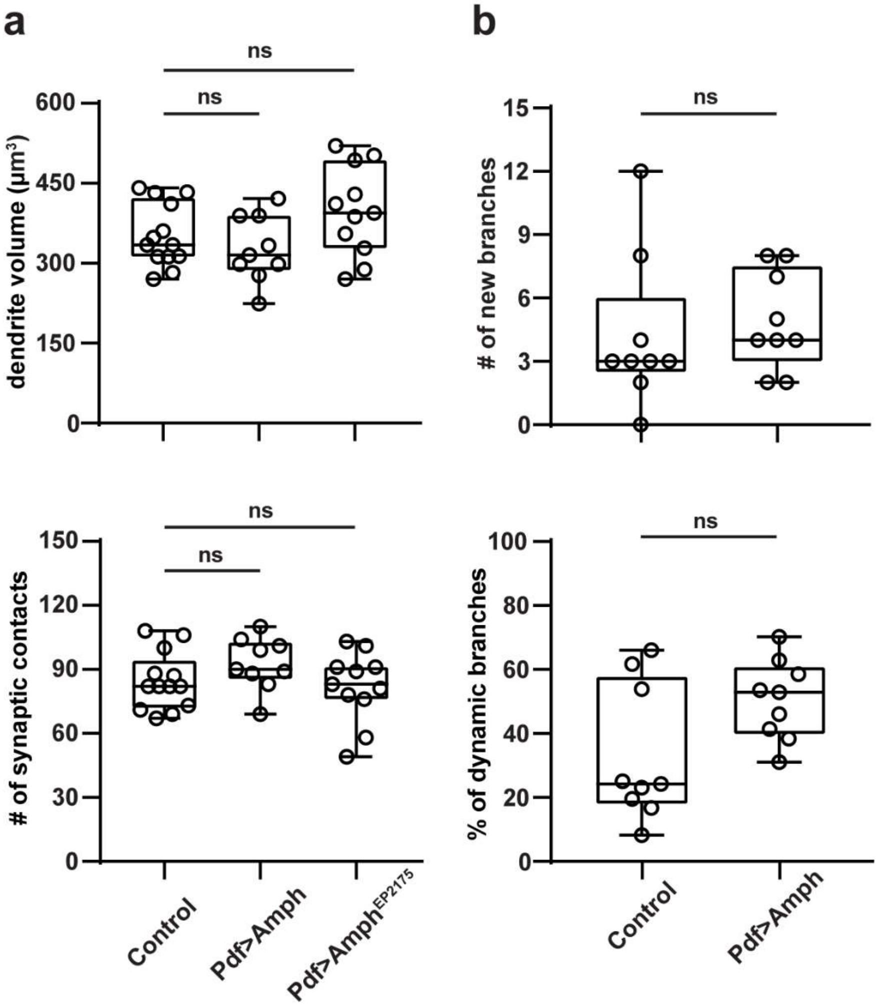

Supplementary Figure 9 Amph overexpression does not affect dendrite volume, synaptogenesis or dendrite dynamics. **a** Amph overexpressed in the LNv through a UAS-Amph transgene, or an EP element in the promoter region, did not change the LNv dendrite volume (top panel) or the number of Brp puncta contacting the LNv dendrites (bottom panel) at 120 hr AEL in LD conditions. n = 13, 9 and 11 for Control, *pdf>Amph* and *pdf>Amph^{EP2175}* respectively. Statistical significance was assessed by one-way ANOVA with Dunnett post hoc test. Volume (top), ANOVA: $F_{2, 30}$ = 2.739, P = 0. 081; Dunnett: P = 0.61, Control vs Pdf>Amph; P = 0.207, Control vs *pdf>Amph ^{EP2175}*. Synapse (bottom), ANOVA: $F_{2, 30}$ = 1.497, P = 0. 24; Dunnett: P = 0.327, Control vs *pdf>Amph*; P = 0.871, Control vs *pdf>Amph^{EP2175}*. **b** Amph overexpressed in the LNv did not change the number of newly formed branches or the percentage of dynamic branches at 120 hr AEL in LL conditions. n = 9 for both groups. Statistical significance was assessed by unpaired *t* test. New branches (top): t_{16} = 0.468, P = 0.647. Dynamic branches: t_{16} = 2.114, P = 0.051. Data are presented as a box plot overlaid with a dot plot; the box plot shows the median (center line in box), interquartile ranges (top and bottom of box, 25–75%) and total ranges (whiskers, 0–100%) and the dot plot displays individual data points. ns, not significant.

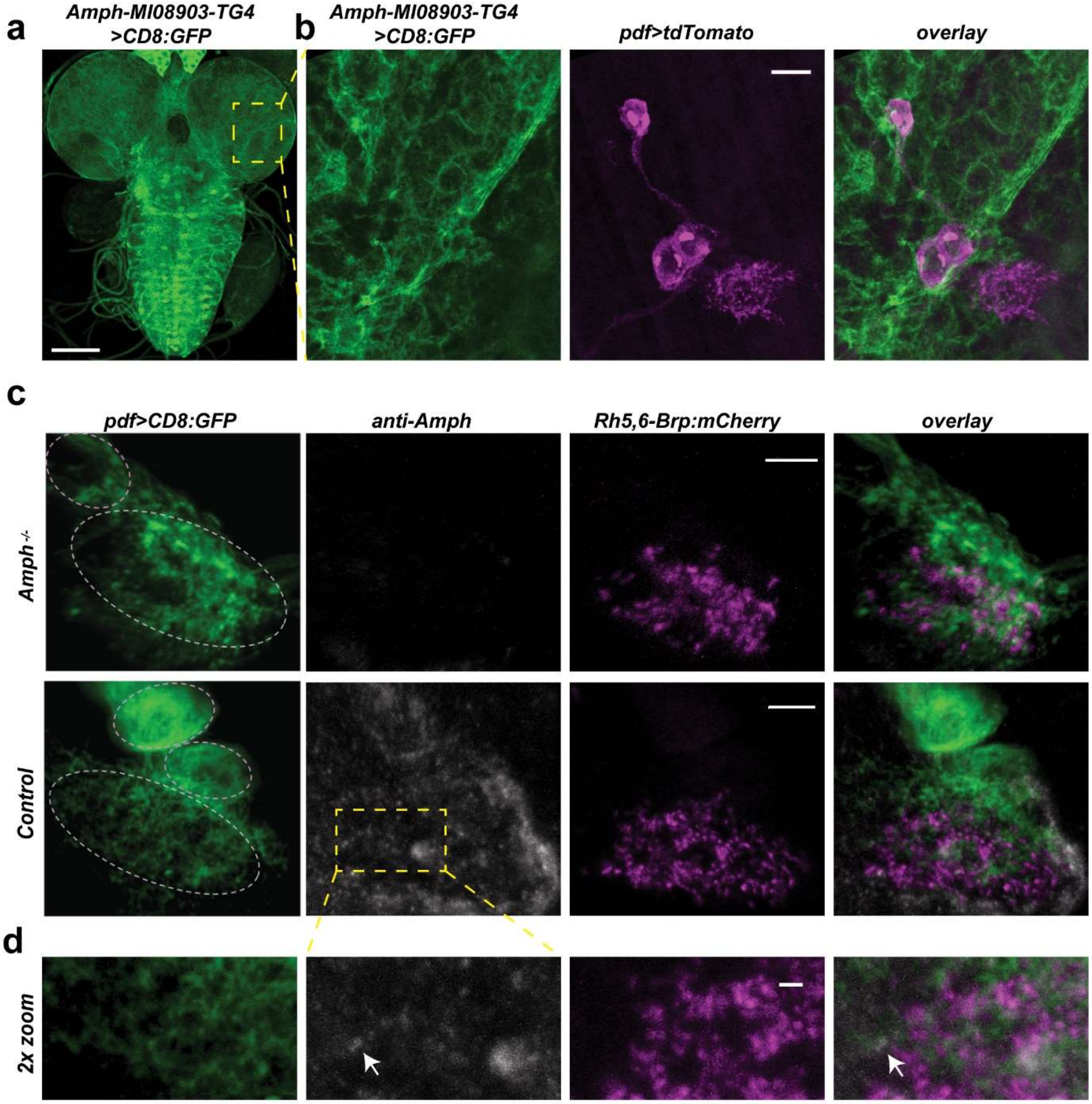

Supplementary Figure 10 Amph is broadly expressed in larval CNS neurons, including the LNvs. **a-b** The broad expression of Amph in the 3rd instar larval brain is visualized by CD8:GFP driven by the Amph-Gal4 ($Amph^{MI08903}$-TG4). Representative confocal images showing the larval CNS (left) and the zoomed in image of the LON (right) are shown. The LNv somas and dendritic region are labeled by pdf-lexA driven lexAop-CD8:tdtomato (magenta). Scale bar, 100 µm in **a**, and 10 µm in **b**. **c** Immunostaining using the anti-Amph antibody (gray) illustrated the diffused distribution of Amph in the LON and in the soma and dendritic region of LNvs (dashed gray outlines). The null mutant showed no signals in the same region. LNvs are visualized by pdf>CD8:GFP (green); the presynaptic terminals from the BN are visualized by Rh5,6-Brp:mCherry (magenta). Scale bar, 5 µm. **d** A single optic section (0.4 µm) of the zoomed in view of the outlined area (yellow) in **c**, showing the localization of Amph signal on the LNv dendrites (arrow), but not overlapping with Brp:mCherry signals. Scale bar, 5 µm.

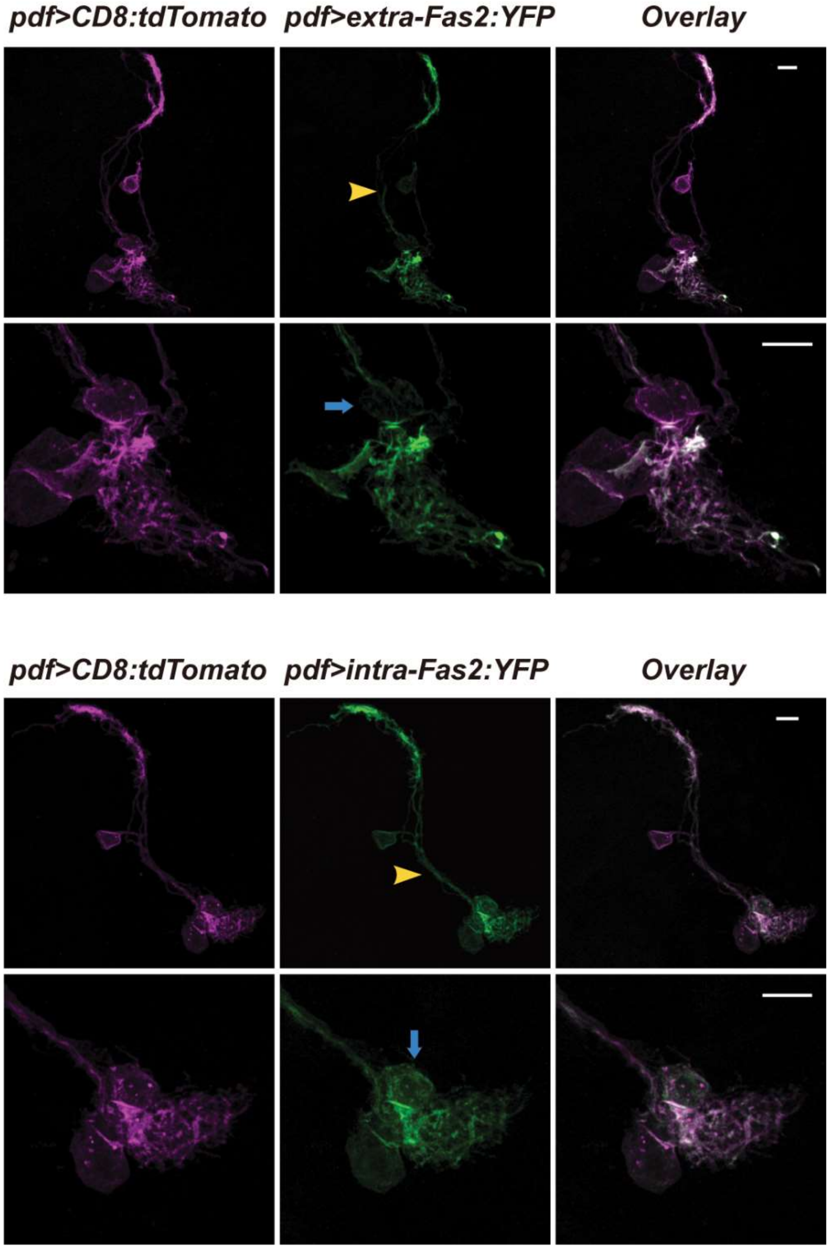

Supplementary Figure 11 Visualization of the dendritic distribution of Fas2 by the YFP fused extracellular domain of Fas2. Representative projected confocal images of neurons (Upper panels) or LNv dendrites (Lower panels) expressing extra-Fas2:YFP or intra-Fas2:YFP are shown. Expressing extra-Fas2:YFP or intra-Fas2:YFP did not alter the neuronal morphology as visualized by *pdf>CD8:tdTomato*. Extra-Fas2:YFP is expressed mainly in dendrite and axonal terminals. Intra-Fas2:YFP is expressed in a diffused pattern and can be found on the plasma membrane. Arrows show the localization of intra-Fas2:YFP on the axonal tract (yellow) and soma membrane (blue), but not extra-Fas2:YFP. Scale bar, 10 μm.

Supplementary Methods

List of fly genotypes by figures

Figure 1, 2 and 4:

hs-flp; pdf-Gal4; UAS-FRT-CD2-stop-FRT-CD8:GFP

Figure 3 and 5:

pdf-Gal4, UAS-CD8:GFP; ; Rh5-Brp:mCherry, Rh6-Brp:mCherry

Figure 6:

Panels a and b: 1). *pdf-Gal4, UAS-CD8:GFP; UAS-Dicer2;* 2). *pdf-Gal4, UAS-CD8:GFP; UAS-Dicer2; UAS-*$Amph^{RNAi}$,

Panel c and d: 1). *hs-flp; pdf-Gal4, UAS-Dicer2; UAS-FRT-CD2-stop-FRT-CD8:GFP* 2). *hs-flp; pdf-Gal4, UAS-Dicer2; UAS-*$Amph^{RNAi}$*, UAS-FRT-CD2-stop-FRT-CD8:GFP*

Panel e: 1). *pdf-Gal4, UAS-CD8:GFP;UAS-Dicer2; Rh5-Brp:mCherry, Rh6-Brp:mCherry* 2). *pdf-Gal4, UAS-CD8:GFP; UAS-Dicer2; UAS-*$Amph^{RNAi}$*, Rh5-Brp:mCherry, Rh6-Brp:mCherry*

Figure 7:

Panel a, b and c:

1). *pdf-Gal4, UAS-CD8:GFP; ; Rh5-Brp:mCherry, Rh6-Brp:mCherry* 2). *pdf-Gal4, UAS-CD8:GFP;* $amph^{5E3}$*; Rh5-Brp:mCherry, Rh6-Brp:mCherry* 3). *pdf-Gal4, UAS-CD8:GFP;* $amph^{5E3}$*; UAS-Amph, Rh5-Brp:mCherry, Rh6-Brp:mCherry*

Figure 8:

Panel b and c: 1). *pdf-Gal4; UAS-Dicer2, UAS-GCaMP6s;* 2). *pdf-Gal4; UAS-Dicer2, UAS-GCaMP6s; UAS-*$Amph^{RNAi}$

Panel d and e: 1). *pdf-Gal4, UAS-CD8:GFP;UAS-Dicer2; Rh5-Brp:mCherry, Rh6-Brp:mCherry* 2). *pdf-Gal4, UAS-CD8:GFP; UAS-Dicer2; UAS-*$Amph^{RNAi}$*, Rh5-Brp:mCherry, Rh6-*

Brp:mCherry 3). *pdf-Gal4; UAS-Dicer2, UAS-extra-Fas2:YFP;* 4). *pdf-Gal4; UAS-Dicer2, UAS-extra-Fas2:YFP; UAS-Amph^{RNAi}*

Panel f -h: 1). *pdf-Gal4; UAS-Dicer2, UAS-extra-Fas2:YFP; Rh5-Brp:mCherry, Rh6-Brp:mCherry;* 2). *pdf-Gal4; UAS-Dicer2, UAS-extra-Fas2:YFP; UAS-Amph^{RNAi}, Rh5-Brp:mCherry, Rh6-Brp:mCherry*

Supplementary Figure 1, 3 and 4:

pdf-Gal4, UAS-CD8:GFP; ; Rh5-Brp:mCherry, Rh6-Brp:mCherry

Supplementary Figure 2 and 5:

hs-flp; pdf-Gal4; UAS-FRT-CD2-stop-FRT-CD8:GFP

Supplementary Figure 6:

Panel a-b: 1). *pdf-Gal4, UAS-CD8:GFP; ; Rh5-Brp:mCherry, Rh6-Brp:mCherry* 2). *pdf-Gal4, UAS-CD8:GFP; UAS-Fas2Δ3; Rh5-Brp:mCherry, Rh6-Brp:mCherry*

Panel c: 1). *hs-flp; pdf-Gal4; UAS-FRT-CD2-stop-FRT-CD8:GFP 2). hs-flp; pdf-Gal4, UAS-Fas2Δ3; UAS-FRT-CD2-stop-FRT-CD8:GFP*

Supplementary Figure 7:

1). *pdf-Gal4, UAS-CD8:GFP; UAS-Dicer2 ; Rh5-Brp:mCherry, Rh6-Brp:mCherry* 2). *pdf-Gal4, UAS-CD8:GFP; UAS-Dicer 2; Rh5-Brp:mCherry, Rh6-Brp:mCherry, UAS-Amph^{RNAi}*

Supplementary Figure 8:

Panel b: *canton-s*

Panel c: 1). *pdf-LexA, LexAop-CD8:tdTomato* 2). *amph^{26}; pdf-LexA, LexAop-CD8:tdTomato*

Supplementary Figure 9:

Panel a: 1). *pdf-Gal4, UAS-CD8:GFP; ; Rh5-Brp:mCherry, Rh6-Brp:mCherry*. 2). *pdf-Gal4, UAS-CD8:GFP; ; UAS-Amph, Rh5-Brp:mCherry, Rh6-Brp:mCherry*. 3). *pdf-Gal4, UAS-CD8:GFP; Amph*^{EP2175}*; Rh5-Brp:mCherry, Rh6-Brp:mCherry*

Panel b: 1). *hs-flp; pdf-Gal4; UAS-FRT-CD2-stop-FRT-CD8:GFP* 2). *hs-flp; pdf-Gal4; UAS-Amph, UAS-FRT-CD2-stop-FRT-CD8:GFP*

Supplementary Figure 10:

Panel a: *amph-MI08903-TG4-Gal4, UAS-CD8:GFP;*

Panel b: *amph-MI08903-TG4-Gal4, UAS-CD8:GFP; pdf-LexA, LexAop-CD8:tdTomato*

Panel c-d: 1). *pdf-Gal4, UAS-CD8:GFP; ; Rh5-Brp:mCherry, Rh6-Brp:mCherry* 2). *pdf-Gal4, UAS-CD8:GFP; amph*^{5E3}*; Rh5-Brp:mCherry, Rh6-Brp:mCherry*

Supplementary Figure 11:

1). *pdf-Gal4; UAS-extra-Fas2:YFP; pdf-LexA, LexAop-CD8:tdTomato* 2). *pdf-Gal4; UAS-intra-Fas2:YFP; pdf-LexA, LexAop-CD8:tdTomato*
