# Supplementary material for: Experience-dependent structural plasticity targets dynamic filopodia in regulating dendrite maturation and synaptogenesis
Source: Nat Commun. 2018 Aug 22;9:3362. doi: 10.1038/s41467-018-05871-5 (PMC6105721; doi:10.1038/s41467-018-05871-5)
Supplement: Supplementary file 3 — Description of Additional Supplementary Files [file 41467_2018_5871_MOESM3_ESM.pdf]

Description of Additional Supplementary Files

File Name: Supplementary Movie 1
Description: A time-lapse live imaging dataset of a single-labeled LNv collected at 48 hr after egg laying (AEL) (Fig. 1b top panel). Ten maximum intensity projected images of the LNv soma and dendrites that are collected at 1 min/frame are played twice at a speed of 2 frames per second. The corresponding time is shown in the top right corner. Genotype: *hs-flp; pdf-Gal4; UAS-FRT-CD2-stop-FRT-CD8:GFP.*

File Name: Supplementary Movie 2
Description: Dendrite dynamics in a single-labeled LNv collected at 48 hr AEL (Fig. 1b bottom panel). 4D tracings of all branch tips (time-stamped traces) are overlaid with time-lapse imaging series of a single LNv labeled by CD8:GFP (red). Ten maximum intensity projected images collected at 1 min/frame are played twice at a speed of 2 frames per second. The corresponding time is shown in the bottom right corner.

File Name: Supplementary Movie 3
Description: Dendrite dynamics in a single-labeled LNv collected at 72 hr AEL (Fig. 1d top panel). 4D tracings of all branch tips (time-stamped traces) are overlaid with time-lapse imaging series of a single LNv labeled by CD8:GFP (red). Ten maximum intensity projected images collected at 1 min/frame are played twice at a speed of 2 frames per second. The corresponding time is shown in the bottom right corner.

File Name: Supplementary Movie 4
Description: Dendrite dynamics in a single-labeled LNv collected at 96 hr AEL (Fig. 1d middle panel). 4D tracings of all branch tips (time-stamped traces) are overlaid with time-lapse imaging series of a single LNv labeled by CD8:GFP (red). Ten maximum intensity projected images collected at 1 min/frame are played twice at a speed of 2 frames per second. The corresponding time is shown in the bottom right corner.

File Name: Supplementary Movie 5
Description: Dendrite dynamics in a single-labeled LNv collected at 120 hr AEL (Fig. 1d bottom panel). 4D tracings of all branch tips (time-stamped traces) are overlaid with time-lapse imaging series of a single LNv labeled by CD8:GFP (red). Ten maximum intensity projected images collected at 1 min/frame are played twice at a speed of 2 frames per second. The corresponding time is shown in the bottom right corner.

File Name: Supplementary Movie 6
Description: Dendrite dynamics in a single-labeled LNv collected at 120 hr AEL from larvae cultured under the light: dark condition (LD) (Fig. 4a top panel). 4D tracings of all branch tips (time-stamped traces) are overlaid with time-lapse imaging series of a single LNv labeled by CD8:GFP (red). Ten maximum intensity projected images collected at 1 min/frame are played twice at a speed of 2 frames per second. The corresponding time is shown in the bottom right corner.

File Name: Supplementary Movie 7
Description: Dendrite dynamics in a single-labeled LNv collected at 120 hr AEL from larvae cultured under the constant light condition (LL) (Fig. 4a middle panel). 4D tracings of all branch tips (time-stamped traces) are overlaid with time-lapse imaging series of a single LNv labeled by CD8:GFP (red). Ten maximum intensity projected images collected at 1 min/frame are played twice at a speed of 2 frames per second. The corresponding time is shown in the bottom right corner.

File Name: Supplementary Movie 8
Description: Dendrite dynamics in a single-labeled LNv collected at 120 hr AEL from larvae cultured under the constant dark condition (DD) (Fig. 4a bottom panel). 4D tracings of all branch tips (time-stamped traces) are overlaid with time-lapse imaging series of a single LNv labeled by CD8:GFP (red). Ten maximum intensity projected images collected at 1 min/frame are played twice at a speed of 2 frames per second. The corresponding time is shown in the bottom right corner.

File Name: Supplementary Movie 9
Description: Dendrite dynamics in a single-labeled LNv collected at 120 hr AEL from larvae cultured under the LD condition (Fig. 6c top panel). The genotype is: *pdf>Dicer2, CD8:GFP.* 4D tracings of all branch tips (time-stamped traces) are overlaid with time-lapse imaging series of a single LNv labeled by CD8:GFP (red). Ten maximum intensity projected images collected at 1 min/frame are played twice at a speed of 2 frames per second. The corresponding time is shown in the bottom right corner.

File Name: Supplementary Movie 10
Description: Dendrite dynamics in a single-labeled LNv collected at 120 hr AEL from larvae cultured under the LD condition (Fig. 6c bottom panel). The genotype is: *pdf>Dicer2,* $Amph^{RNAi}$; *CD8:GFP.* 4D tracings of all branch tips (time-stamped traces) are overlaid with time-lapse imaging series of a single LNv labeled by CD8:GFP (red). Ten maximum intensity projected images collected at 1 min/frame are played twice at a speed of 2 frames per second. The corresponding time is shown in the bottom right corner.

File Name: Supplementary Data 1
Description: The table lists the 134 genes and their corresponding RNAi lines tested in a dendrite dynamics RNAi screening. A stock number started with a v indicates that it is from Vienna Drosophila Resource Center (VDRC). The rest are from Bloomington Drosophila Stock Center (BDSC). The last column indicates the phenotype generated by the transgenic RNAi on LNv dendrite dynamics in 3rd instar larvae cultured under the constant light conditions. The phenotype was accessed by visual inspections.
